# Antibiotics, gut microbiota, and Alzheimer’s disease

**DOI:** 10.1186/s12974-019-1494-4

**Published:** 2019-05-22

**Authors:** Francesco Angelucci, Katerina Cechova, Jana Amlerova, Jakub Hort

**Affiliations:** Memory Clinic, Department of Neurology, 2nd Faculty of Medicine, Charles University and Motol University Hospital, Prague, Czech Republic

**Keywords:** Alzheimer’s disease, Gut microbiota, Antibiotics, Neuroinflammation

## Abstract

Alzheimer’s disease (AD) is a neurodegenerative disease whose various pathophysiological aspects are still being investigated. Recently, it has been hypothesized that AD may be associated with a dysbiosis of microbes in the intestine. In fact, the intestinal flora is able to influence the activity of the brain and cause its dysfunctions.

Given the growing interest in this topic, the purpose of this review is to analyze the role of antibiotics in relation to the gut microbiota and AD. In the first part of the review, we briefly review the role of gut microbiota in the brain and the various theories supporting the hypothesis that dysbiosis can be associated with AD pathophysiology. In the second part, we analyze the possible role of antibiotics in these events. Antibiotics are normally used to remove or prevent bacterial colonization in the human body, without targeting specific types of bacteria. As a result, broad-spectrum antibiotics can greatly affect the composition of the gut microbiota, reduce its biodiversity, and delay colonization for a long period after administration. Thus, the action of antibiotics in AD could be wide and even opposite, depending on the type of antibiotic and on the specific role of the microbiome in AD pathogenesis.

Alteration of the gut microbiota can induce changes in brain activity, which raise the possibility of therapeutic manipulation of the microbiome in AD and other neurological disorders. This field of research is currently undergoing great development, but therapeutic applications are still far away. Whether a therapeutic manipulation of gut microbiota in AD could be achieved using antibiotics is still not known. The future of antibiotics in AD depends on the research progresses in the role of gut bacteria. We must first understand how and when gut bacteria act to promote AD. Once the role of gut microbiota in AD is well established, one can think to induce modifications of the gut microbiota with the use of pre-, pro-, or antibiotics to produce therapeutic effects.

## Introduction

Alzheimer’s disease (AD) is a neurodegenerative disease whose various pathophysiological aspects are still under investigation [1]. It is a disorder characterized by a progressive decline in cognitive functions and loss of specific types of neurons and synapses. The most recognized pathological events in AD are amyloid plaques and neurofibrillary tangles [2]. Amyloid plaques are extracellular accumulations of abnormally folded amyloid beta (Aβ) proteins with 40 or 42 amino acids (Aβ40 and Aβ42), two by-products of amyloid precursor protein (APP) metabolism [3]. Neurofibrillary tangles are primarily composed of paired helical filaments consisting of hyperphosphorylated tau, a protein stabilizing microtubules [3]. The etiology of AD is multifactorial. There exist sporadic forms and familial forms associated with mutations in three genes: APP, presenilin 1 (PSEN1), and presenilin 2 (PSEN2). Familial forms are more rare (< 0.5%) as compared to sporadic forms [1]. Nowadays, it is believed that genetic and environmental factors interact to induce AD onset.

Recently, it has been hypothesized that AD may be associated with a dysbiosis of microbes in the intestine [4]. This hypothesis is linked to the fact that the intestinal flora is able to influence the activity of the brain and cause its dysfunctions [2, 5]. Growing evidence in this field led to the definition of the term microbiota-gut-brain axis (MGBA) [6]. The association between gut microbiota and AD is also related to the central role of inflammation in the development and course of AD [7]. Given the growing interest in this topic, the purpose of this review is to analyze the role of antibiotics in relation to the gut microbiota and AD.

### Gut microbiota

Thousands of species of microbes that influence the physiology and development of the individual, as well as the maintenance of the host’s health, populate our intestine (or gut). Among gut microbes, there can be distinguished bacteria, viruses, and fungi. In a healthy organism, these microorganisms regulate the digestive pH and, in turn, create a protective barrier against the infectious agents.

These “good” microbes are called probiotic: a living microorganism that produces beneficial effects on the health of the host person [8]. Probiotic bacteria contribute to make the necessary substances available to our body, to avoid inflammation and related diseases. The whole chain of reactions favorable to our health occurs only when the intestinal bacterial flora is in equilibrium. To favor this equilibrium, it is necessary to consume enough quantity of these probiotics trough the diet. The most common ones are *Bifidobacteria* and *Lactobacillus* strains. They are found in some type of food such as yogurt, fermented cheese, and vegetables, or they can be consumed as dietary supplements. A good variety of microbiota strain can be achieved by a large variety diet, including the habit to consume other types of food during traveling. However, poor eating habits, antibiotic consumption, and stress can compromise their activity and/or alter their composition, creating an imbalance that puts health at risk. The diseases associated to an alteration of the gut microbiota are varied and include colorectal cancer, metabolic syndrome, obesity, allergies, inflammatory bowel disease, type 2 diabetes, and heart failure [9].

### Gut microbiota and brain

The relationship between the gut microbiota and the central nervous system is because the intestine and the brain can interact with each other through the nervous system or chemical substances crossing the blood-brain barrier. In particular, the vagus nerve connects intestinal neurons with those of the central nervous system [10]. The gut microbiota produces substances (i.e., monoamines and amino acids) that, through the lymphatic and vascular system, reach the central neurons and can influence their activity, with possible repercussions on behavior [11]. In addition, gut bacteria are receptive to the messages sent by the brain in the form of neurotransmitters [7, 12].

Several pathways of communication between the gut and brain have been studied [13]. Vagus nerve serves as a link between the gut and the spinal cord (autonomic nervous system) [14]. The vagus nerve ends to brain stem nuclei that receive and give afferent and efferent fibers [14]. In this way, brain stem nuclei may control many gut functions and send signals to other brain regions, such as the thalamus and cortical areas [15]. In addition, the enteric nervous system can exchange signals with the central nervous system through the gut bacteria [16]. Exchanges between gut and brain can also occur through the blood circulation [17]. Intestinal mucosa and blood-brain barriers allow the passage of immune and endocrine molecules, such as cytokines and hormones, able to influence both gut and brain functions [18]. Interestingly, it has been shown in germ-free mice that gut bacteria influence the maturation of the immune, endocrine, and nervous system [15]. The MGBA can be seen as a multifunctional network, where central, peripheral, immune, and endocrine systems participate to the bidirectional communication [19].

The way in which the gut microbiota regulates MGBA can be of various kinds. First, these microorganisms are able to synthesize and release neurotransmitters and neuromodulators, such as short-chain fatty acids (SCFAs), biogenic amines (e.g., serotonin, histamine, and dopamine), and other amino acid-derived metabolites such as serotonin or GABA and tryptophan [13]. All these molecules act as neurotransmitters or as neurotransmitter precursors in the brain and regulate neuronal activity. Nonetheless, there is still the need for more robust experimental evidences to prove that gut microbiota alterations are responsible for changes in behavior. Many studies indeed evidenced this correlation but did not prove a direct cause-effect [20].

Another possibility is that the gut microbiota produces toxic substances to the brain. The gut microbiota can release neurotoxic substances, such as d-lactic acid and ammonia [21]. Moreover, during a process of inflammation, the gut microbiota releases other proteins potentially harmful to the brain, such as proinflammatory cytokines and other innate immune activators in the host [22]. Thus, the microbiota can affect the MGBA via immunological, neuroendocrine, and direct neural mechanisms [17]. The result of this alteration in the brain can lead to memory impairment, anxiety, and other cognitive dysfunctions [20, 21, 23, 24]. According to the recent studies, changes in gut microbiota are associated to various neurological diseases [25], which include not only anxiety and depression [26], but also neurodegenerative diseases [6] or drug-resistant epilepsy [27]. Among neurodegenerative diseases, there are evidences for a possible involvement of gut dysbiosis in AD [4], Parkinson’s [28] and Huntington’s [29] diseases, and multiple sclerosis [30].

### Alzheimer’s disease: the role of inflammation

The connection between the gut microbiota and AD was hypothesized because of the role of inflammation in this pathology [7]. The brain is able to initiate an immune response following different insults, such as pathogens or any other harmful event. Under normal conditions, this immune response is initiated by microglia and terminates with the elimination of pathogens, dead cells or other cellular debris, and tissue restoration. However, under certain pathological conditions in which the insult persists or the immune response is altered or compromised, a process of chronic inflammation can be harmful to neurons. The term “neuroinflammation” refers to the fact that the neurons release substances that sustain the inflammatory process and the immune response. The immune responses can therefore be beneficial or detrimental to the brain, depending on the strengths of their activation.

A prolonged neuroinflammatory process has been shown to be the cause or consequence of some neurodegenerative diseases [31] including AD [32]. In particular, elevated serum levels of proinflammatory cytokines such as interleukin (IL)-1 and IL-6, TNF-alpha, and TGF-beta, which have a central role in neuroinflammation, have been observed in AD patients [33, 34]. The constant release of cytokines by microglia and astrocytes seems to be due to the continuous deposition of the Aβ peptide in the extracellular space [32, 34]. According to the amyloid cascade hypothesis, these deposits lead to the synaptic dysfunction and underlie the clinical symptoms of dementia observed in AD. Nonetheless, this hypothesis has been challenged by repeated failures of clinical trial with Aβ-targeting drugs [35]. It has become evident that Aβ dyshomeostasis is upstream of alterations in other proteins and diverse cell types that contribute to the AD cognitive phenotype. The role of microglia activation, in response to Aβ deposition, has emerged as an important factor in AD pathogenesis [36, 37]. Some genes encoding for proteins of the innate immune response have been identified as a key element of AD pathophysiology. Among them, complement receptor 1 [38], CD33 [39], and TREM2 [40] appear to be involved either directly or indirectly in the response of microglia to Aβ deposition. As shown in transgenic animal models, alterations of these genes lead to a dysfunctional response of microglia, which fail to cluster around Aβ plaques [40–42].

In addition, recent data indicate that Aβ itself, although it was thought to be a proinflammatory peptide [26, 43], appears to have an innate antimicrobial activity [44]. These data suggest that neuroinflammatory processes may be the cause, and not the consequence, of the neurodegenerative processes of AD. Nonetheless, it is yet not clear whether inflammation is the primary event in AD as many studies have shown that Aβ deposition may precede microgliosis [45, 46]. The latest hypotheses suggest that a vicious cycle between Aβ accumulation and microglia activation is present in the brain of AD patients [46] and that microglia-induced neuroinflammation may be a target for anti-AD drug development [47].

In this context, the idea has developed that an alteration of the gut microbiota, a condition called dysbiosis, may be one of the factors contributing to the neuroinflammatory processes observed in AD [48].

### Dysbiosis as an inducing factor in AD

Many studies in recent years have highlighted the role of gut microbiota in AD pathophysiology [4, 49]. Some theories based on a role of gut microbiota have been proposed, including a direct action of these microbes (microbial infection in AD) [50], indirect actions (antimicrobial protection hypothesis, hygiene hypothesis) [29, 31, 49, 51], and processes related to the aging of the immune system [52].

### Direct microbial infection in AD

The demonstration that the gut microbiota is able to participate in AD pathophysiology comes primarily from studies in laboratory animals. In this regard, studies with rodent-free pathogens, the so-called germ-free, are important. In these animals, a significant reduction of the Aβ pathology was observed, which is present again when the mice are exposed to the gut microbiota of the control mice [53].

In humans, many studies have also recently shown that a viral or bacterial infection can be one of the triggering causes of AD. It has been shown that chronic *Helicobacter* (*H.*) *pylori* infection in AD patients triggers the release of inflammatory mediators and is associated with decreased MMSE score as compared to non-infected patients [54]. Moreover, the serum levels of Aβ40 and Aβ42 are higher in AD patients infected by *H. pylori* and other bacteria, such as *Borrelia burgdorferi* and *Chlamydia pneumoniae* [55]. In neuroblastoma cells, it was also demonstrated that exposure to *H. pylori* filtrate induces a tau hyperphosphorylation resembling that observed in AD tau pathology [56].

All these bacteria can act synergistically to induce an infection burden in the brain of AD patients [57]. In hippocampal and temporal lobe lysates from AD brains, high levels of bacterial lipopolysaccharide were observed [58]. Analysis of blood in patients with brain amyloidosis and cognitive impairment also revealed increased levels of proinflammatory cytokines, together with higher proinflammatory (*Escherichia*/*Shighella*) and reduced anti-inflammatory (*Escherichia rectale*) gut microbes [59]. A viral infection was also hypothesized in AD [50]. In particular, many studies have shown that herpes simplex virus type 1 (HSV1) represents an important risk factor for the development of the disease, especially for ApoE-ε4 carriers [60]. Other viruses, such as *Cytomegalovirus* (CMV) [61] and varicella-zoster virus [62], have also been associated with AD, although the role of these viruses as individual AD risk factors is not clear [63, 64].

Brain alterations caused by dysbiosis that can promote AD can occur in many ways. First, as already mentioned, these bacteria are responsible for possible alterations in the levels of certain neurotransmitters. In addition, some studies have shown that gut microbiota can also alter proteins and receptors involved in synaptic plasticity [65], such as NMDA receptors, brain-derived neurotrophic factor (BDNF), and serotonin receptors, in addition to serotonin itself. Inflammation also plays a fundamental role. Dysbiosis can generate a neuroinflammatory state with the production of proinflammatory cytokines and the loss of immune regulatory function [66]. Furthermore, under normal conditions, the gut microbiota is responsible for the production of neuroprotective molecules such as fatty acids and antioxidants [67, 68].

### Age-related dysbiosis and AD

The clinical and experimental evidences of a link between gut microbiota and AD have led to the so-called theory of “age-related dysbiosis,” which hypothesizes that AD may arise during the process of aging of the immune system. In fact, it has been observed that during the aging, there are changes in the composition of the gut microbiota, the increase of proteobacteria, and a reduction of probiotics, such as bifidobacteria, and neuroprotective molecules, such as SCFAs [38, 69]. Moreover, an association between the loss of microbiome function, specifically genes that encode SCFAs, and increased levels of circulating proinflammatory cytokines has been shown in healthy elderly people [70].

It has been suggested that the processes of age-related dysbiosis and neurological decline are linked through the former mediating chronic low-grade inflammation as a common basis for a broad spectrum of age-related pathologies, or so-called inflamm-aging [71].

### Antimicrobial protection in AD

In line with these findings, the hypothesis of antimicrobial protection in AD was postulated [51]. According to this theory, the accumulation of Aβ in the brain is an epiphenomenon that represents an immune response to the accumulation of harmful bacteria. This theory is supported by numerous data that indicate that the peptide Aβ represents a natural antimicrobial agent but, during the AD course, the protracted neuroinflammatory state caused by the gut microbiota leads to a non-interruption of this process, with consequent Aβ accumulation of brain [51].

At the same time, however, it should be noted that the complete absence of gut microbiota is detrimental to the functioning of the brain. If we destroy the bacterial flora using antibiotics in animal models of AD, we can see a reduction in Aβ deposits but also an increase in inflammatory molecules such as cytokines and chemokines and an activation of microglia [72]. Thus, a simple reduction of gut microbiota can be deleterious.

### Hygiene hypothesis of AD

With this in mind, the hygiene hypothesis of AD has been proposed. The hygiene hypothesis of AD points to the excessive sanitation in early life as the cause of subsequent disturbances of the components of the immune system [29, 49]. In this regard, it has been observed that the microglia of germ-free animals seem to be less reactive to inflammatory processes caused by viruses and bacteria, and generally have a reduced, or at least altered, basal surveillance level [73]. The hygiene hypothesis of AD predicts the negative correlation with microbial diversity and is positively associated with environmental sanitation [74].

Dysfunction of the immune system induced by inadequate stimulation to immunity may result in an increased risk of AD through T cell system [75]. Some interesting studies suggest that the functionality of regulatory T (Treg) cells, fundamental elements of Th1-mediated inflammation, is impaired in AD patients and that mild cognitive impaired (MCI) patients have not only a high number of Treg cells as compared to controls [76] but also a higher Treg-induced immunosuppression [77]. In addition, inadequate Treg function in these patients augments the risk of conversion from MCI to AD [78] while individuals with adequate Treg function may stay longer in the MCI phase [79].

These data highlight the importance of immune cell components in the development of AD and further support the hygiene hypothesis. In addition, some studies have shown that subjects carrying genes of AD familiar forms, such as apolipoprotein E (ApoE)-4 allele carriers, present an increased risk of AD conversion in the presence of viral infections [49, 80] or food regimes [50, 81] harmful to gut bacteria.

In conclusion, any element that disturbs the intestinal flora and its balance can be a triggering factor for neurological disorders, including AD, especially during the old age where the immune defenses are lacking or are reduced. Among these elements, we can include not only microbial infections but also other factors, such as diet and the use of antibiotics.

### Antibiotics, gut microbiota, and Alzheimer’s disease

If the gut microbiota plays an important role in AD, substances that are able to modify its composition, such as antibiotic agents, can positively or negatively affect the disease. Antibiotics are normally used to remove or prevent bacterial colonization in the human body, without targeting specific types of bacteria. As a result, broad-spectrum antibiotics can greatly affect the composition of the gut microbiota, reduce its biodiversity, and delay colonization for a long period after administration.

A number of studies showed that different antibiotic treatments result in short- and/or long-term changes in the intestinal microbiota in both humans and animals [82]. In addition, both animal and clinical studies have demonstrated that the use of antibiotics and the concomitant dysbiosis is associated with changes in behavior and brain chemistry [83, 84].

In humans, it has been demonstrated that antibiotic use, when administered as a cocktail therapy, is associated with neurological disorders that include anxiety and panic attacks to major depression, psychosis, and delirium [85]. Despite this, the normal use of antibiotics in the general population is not typically associated with neuropsychiatric side effects. With regard to AD, it has been shown that the use of cocktail of antibiotics (ABX) in APP/PS1 transgenic mice can increase the neuroinflammatory state and cytokine levels and therefore the disease itself [72].

Among the harmful antibiotics, there are those that destroy the balance of gut bacteria, such as streptozotocin and ampicillin [86]. According to the hypotheses on gut microbiota and AD, the use of these antibiotics favors the disease or worsens its course. The administration of ampicillin in rats produced an elevation of serum corticosterone and increased the anxiety-like behavior and impairment of spatial memory [87]. Elevated glucocorticoids are associated with memory dysfunctions and reduction of hippocampal BDNF, two common features of AD pathology. Interestingly, the administration of probiotics (*Lactobacillus fermentum* strain NS9) reverses the physiological and psychological abnormalities induced by ampicillin in rats [87]. In this regard, germ-free mice are also characterized by similar molecular alterations, such as of anxiety-like behavior [88] and changes in the expression of tight junction proteins, BDNF [89], GRIN2B, the serotonin transporter, the NPY system [84], and HPA axis activity.

It has been also demonstrated that NMDA receptor expression might be dependent on the presence of gut microbiota. The mRNA expression of hippocampal NMDA receptor subtype 2B (NR2B) is significantly decreased in germ-free mice [88]. Disruption of gut microbiota by ampicillin treatment also significantly reduces the level of NMDA receptor in the rat hippocampus [87].

Further support to this notion is the fact that antibiotics such as streptozotocin have been used to induce sporadic AD forms in animal models with effects on learning and memory performances [59, 90]. The same antibiotic is used to induce diabetes mellitus in animals [60, 91] which is a frequent comorbidity of AD characterized by cognitive decline [61, 92]. Moreover, the administration of probiotic substances as a food supplement has beneficial effects on the synaptic activity and cognitive function in streptozocin-induced diabetes rat models [93].

In line with the hygiene hypothesis of the disease, there is evidence that the administration of antibiotic cocktails in adolescent mice can cause permanent alterations of the gut microbiota and increase in proinflammatory cytokines, with long-lasting effects on cognitive function in the adult [94, 95]. In humans, some antibiotics, i.e., cefepime, can cross the blood-brain barrier and cause altered mental status, with reduced consciousness, myoclonus, and confusion [65, 96], without the mediation of gut microbiota. On the other hand, antibiotics can also have beneficial effects on AD. These effects are due to the fact that an alteration of the gut microbiota, not necessarily caused by antibiotics, can promote the development of bacteria that could be harmful to the brain (microbial hypothesis) [24]. The elimination of pathogenic bacteria such as *Helicobacter pylori* by triple eradication antibiotic regimen (omeprazole, clarithromycin, and amoxicillin) has led to positive results for cognitive and functional status parameters in AD patients [97].

A series of studies have also shown that some antibiotics, by reducing neuroinflammation due to dysbiosis, can have beneficial effects in AD. These effects include neuroprotection and anti-inflammatory, anti-tau, anti-amyloid, and cholinergic effects. The administration of rifampicin in AD animal models reduces the brain levels of Aβ and inflammatory cytokines [98]. Minocycline has also similar effects on Aβ and reduces microglia activation in rodent AD models [99]. Similarly, rapamycin has been shown to reduce not only Aβ and the microglia activation, but also tau phosphorylation [100]. d-Cycloserin, which is also a NMDA receptor partial agonist, improves cognitive deficits in aged rats [101] and in AD patients [102].

All these antibiotics have been proved to reduce inflammation and improve cognitive deficits in AD animal models, while controversial results have been obtained in some clinical trials.

In 2004, doxycycline and rifampin given in combination showed a significant improvement in the Standardized Alzheimer’s Disease Assessment Scale cognitive subscale (SADAScog) at 6 months in patients with probable AD and mild to moderate dementia [103]. In 2013 instead, a multicenter, blinded, randomized, 2 × 2 factorial controlled trial in patients with mild to moderate AD showed no significant effect on cognition after 12 months of treatment with doxycycline or rifampin, alone or in combination [104]. Similarly, in 1999, d-cycloserine was found effective in improving cognitive deficits in patients with AD [102] but these positive effects were not replicated in successive trials [105]. The presence or absence of bacterial infections, like *H. pylori* [97], susceptible to antibody action may be responsible for these contrasting data. Nonetheless, these studies provide evidence for a possible role of antibodies in AD through their action on gut bacteria.

In addition, besides contrasting neuroinflammation [99], antibiotics can also have beneficial effects in AD through other mechanisms. This is the case of rapamycin, which, in addition to having so-called antiaging properties [106], is in fact the natural inhibitor of the mammalian enzyme target of rapamycin (mTOR). Upregulation of the mTOR signaling pathway plays an important role in major pathological processes of AD. The administration of mTOR inhibitors, like rapamycin, ameliorate the AD-like pathology and cognitive deficits in a broad range of animal models [100], indicating their potential as therapeutics.

Despite these findings, the option to use antibiotics to treat AD and other neurodegenerative disorders should be carefully evaluated in humans. The possible benefits can be counteracted by the insurgence of antibiotic resistance. At present, there is lack of scientific evidences for the use of antibiotics as therapeutic agents for AD.

### Probiotics, prebiotics, and Alzheimer’s disease

Probiotics are bacteria that have beneficial effects on the health of the host person [8] while prebiotics are substances (mostly fiber) which serve as food for these bacteria. The data on the effects of probiotics (and prebiotics) in AD are not yet abundant. Some studies have investigated the effect of certain types of diet in humans. The results demonstrated that healthy dietary patterns characterized by high intake of probiotics and prebiotics, in association to other nutrients, delay neurocognitive decline and reduce the risk of AD [107]. In addition, it was shown that probiotic diet supplementation not only has an effect on normal brain activity [108] but also induces significant cognitive improvements in AD patients [109]. These effects may be due to the restoration of gut microbiota, but also to the contrasting action to other AD-related pathological events, such as oxidative stress and insulin resistance [109, 110]. More recently, it has been demonstrated that transgenic AD mice treated with probiotics, as compared to untreated AD mice, have better cognitive performance and reduced number of Aβ plaques in the hippocampus [111]. Similar effects on cognitive function in AD transgenic mice have been reported after prebiotic administration [112]. Finally, as stated before, probiotic administration in rats reverses the physiological and psychological alterations induced by administration of the antibiotic ampicillin [87].

## Conclusions: antibiotics or probiotics as AD therapies?

**Fig. 1:**
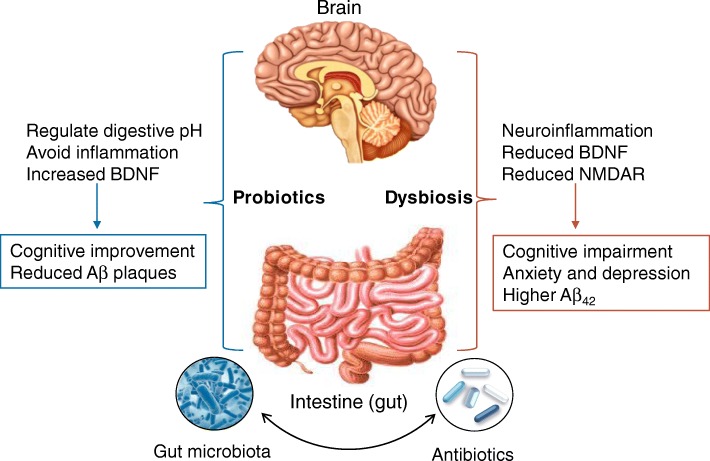
Schematic representation of the role of microbiota-gut-brain axis in Alzheimer’s disease. Good bacteria probiotics are capable to stabilize digestive pH, reduce inflammation, and increase neuroprotective molecules, such as brain-derived neurotrophic factor (BDNF). These effects lead to improved cognition and reduced Aβ plaque formation in AD animal models. In contrast, impaired microbiota dysbiosis can induce neuroinflammation and reduce the expression of BDNF and NMDA receptor, leading to cognitive impairment, mood disorders, and higher levels of Aβ_42_. Antibiotics, by affecting gut microbiota composition, interact with this circuit and produce different effects, depending on their microbiome target

As described above, alteration of the gut microbiota can induce changes in brain activity, which raises the possibility of therapeutic manipulation of the microbiome in AD and other neurological disorders (Fig. 1). The possibility of a therapeutic, or preventive, intervention using antibiotics in AD is intriguing because of the cost benefits of such treatments, which could be relatively inexpensive and can be combined with specific dietary regimen with probiotics to act synergistically. This field of research is currently undergoing great development, but therapeutic applications are still far away. Whether a therapeutic manipulation of gut microbiota in AD could be achieved using antibiotics or probiotics is still not known. The action of antibiotics in AD could be wide and even opposite, depending on the type of antibiotic (Table 1) and on the specific role of the microbiome in AD pathogenesis.

**Table 1:** Cited studies on the effects of antibiotics in AD rodent models and humans

Antibiotic	Species	Target	Effects	References
Streptozotocin	-Mice -Rats	-Gram-positive bacteria -Pancreatic islet cells	-Memory deficits	[90, 93]
Ampicillin	-Rats	-Gram-positive and Gram-negative bacteria	-Increased serum corticosterone -Increased anxiety -Memory deficits	[87]
Cefepime	-Humans	-Gram-positive and Gram-negative bacteria	-Reduced consciousness, myoclonus, confusion	[96]
Amoxicillin	-Humans	-Gram-positive bacteria	-Improved cognition	[97]
Rifampicin	-Humans -Rats -Mice	-Bacterial DNA-dependent RNA synthesis	-Anti-cholinesterase -Anti-oxidative -Anti-inflammatory -Reduced Aβ	[98, 102, 103]
Minocycline	-Mice -Rats	-Gram-positive and Gram-negative bacteria	-Reduced inflammation and microglia activation -Improved cognition -Reduced Aβ	[99]
Rapamycin	-Mice -Rats	-Antifungal -Immunosuppressant -mTOR inhibitor	-Improved cognition -Reduced tau -Reduced Aβ -Reduced microglia activation	[100, 105]
d-Cycloserin	-Humans -Rats	-Gram-positive and Gram-negative bacteria -NMDA receptor partial agonist	-Improved cognition	[101, 104]
Doxycycline	-Humans -Mice	-Gram-positive and Gram-negative bacteria	-Improved cognition -Reduced inflammation	[102, 103]

As emerged from the studies mentioned, the use of antibiotics against gut microbiota specifically related to AD may be useful. The elimination of chronic infections caused by *H. pylori* or HSV1 virus can bring benefits to disease prevention, but also positive effects on cognitive functions. Nonetheless, clinical trials with antibiotics on patients already suffering from AD have led to conflicting results. Among the main problems, we must consider the multifactorial nature of the disease, which can be associated to an inflammatory state, but not exclusively. The presence of *H. pylori* infection, for example, may influence the outcome of a clinical trial, as its elimination may lead to cognitive improvements in affected patients, but it may prove ineffective in unaffected patients. Furthermore, there is always a real risk of causing dysbiosis in an attempt to reduce a state of neuroinflammation. Many antibiotics have a broad and not selective action on certain pathogens. In addition, other factors can affect the composition of the gut microbiota. Among these, diet [113, 114], alcohol consumption [115], smoking [116], and changes in circadian rhythm [117] have been shown to affect the microbiota composition. The negative effects of antibiotics can be contrasted by the concomitant treatment with probiotics. Nevertheless, the development of antibiotics with selective antimicrobial action is desirable. A crucial factor is therefore the identification of the gut microbiota associated with the disease. At present, there are no definitive data on which types of gut microbiota are altered in AD. Thus, the future of antibiotics as therapeutics in AD depends on the research progresses in the role of gut microbiota.

Preclinical studies can certainly help to answer these questions. The manipulation of germ-free animals with various bacterial strains present in gut microbiota could provide specific indications on the possible therapeutic targets related to AD. At that point, one can think of inducing gut microbiota modifications with the use of pre-, pro-, or antibiotics to obtain beneficial effects.

## Abbreviations

AD: Alzheimer’s disease
ApoE: Apolipoprotein E
Aβ: Amyloid beta
BDNF: Brain-derived neurotrophic factor
GRIN2B: Glutamate ionotropic receptor NMDA type subunit 2B
HPA: Hypothalamic-pituitary-adrenal
IL: Interleukin
MCI: Mild cognitive impairment
MGBA: Microbiota-gut-brain axis
mTOR: Mammalian target of rapamycin
NMDA: *N*-Methyl-d-aspartate
NPY: Neuropeptide Y
NR2B: *N*-Methyl-d-aspartate receptor subtype 2B
SCFAs: Short-chain fatty acids
TDP-43: TAR DNA-binding protein 43
TGF-beta: Transforming growth factor beta
TNF-alpha: Tumor necrosis factor alpha
Treg: Regulatory T
